# The Biocompatibility and Self-Healing Effect of a Biopolymer’s Coating on Zn Alloy for Biomedical Applications

**DOI:** 10.3390/ma16237486

**Published:** 2023-12-02

**Authors:** Katarzyna Cesarz-Andraczke, Badegül Tuncay, Wojciech Pakieła, Zbigniew Brytan, Magdalena Skonieczna, Jana Bidulská, Robert Bidulsky

**Affiliations:** 1 Department of Engineering Materials and Biomaterials, Faculty of Mechanical Engineering, Silesian University of Technology, 44-100 Gliwice, Poland; wojciech.pakiela@polsl.pl (W.P.); zbigniew.brytan@polsl.pl (Z.B.); 2 Department of Mechanical Engineering, Karabuk University, 78050 Karabuk, Turkey; badegultuncay@gmail.com; 3 Department of Systems Biology and Engineering, Faculty of Automatic Control, Electronics and Computer Science, Silesian University of Technology, 44-100 Gliwice, Poland; magdalena.skonieczna@polsl.pl; 4 Department of Plastic Deformation and Simulation Processes, Institute of Materials and Quality Engineering, Faculty of Materials, Metallurgy and Recycling, Technical University of Kosice, Park Komenského 11, 04001 Kosice, Slovakia; 5 Bodva Industry and Innovation Cluster, Budulov 174, 04501 Moldava nad Bodvou, Slovakia; director@biic.sk

**Keywords:** self-healing effect, resorbable biomaterials, zinc alloys, biocompatibility, casein

## Abstract

The objective of this study was to formulate dip coatings, incorporating casein, NaOH, and nanocrystalline hydroxyapatite (nanoHAp), with self-healing properties for application on ZnMg3.2 wt.% alloy in the field of biomedical applications. This study hypothesizes that the self-healing mechanism within the layer will impede substrate degradation by progressively filling defects where chlorides from simulated body fluids intervene. Furthermore, it aims to mitigate potential damage effects during the implantation process by the layer’s self-healing capabilities. The research focused on the dip-coating process parameters and chemical composition of baths for producing casein coatings on Zn alloy surfaces. This study investigated the impact of casein and NaOH concentration, along with the immersion time of ZnMg3.2 wt.% samples in the coating bath, on the self-healing capability of the coating under simulated human body fluid conditions (Ringer’s solution, temperature: 37 °C). Effective technology was developed by selecting specific chemical compositions and immersion times in the coating bath, enhancing the self-healing progress against coating damage in Ringer’s solution at 37 °C. The most significant self-healing effect was observed when the ZnMg3.2 wt.% substrate underwent a 1 h immersion in a coating bath containing 2 g of casein, 4 g of NaOH, and 0.1 g of nanoHAp powder. Electrochemical tests were instrumental in determining the optimal casein concentration and immersion time of the Zn alloy in the coating bath.

## 1. Introduction

The most common threat to the health of modern society includes osteoarticular system injuries, as well as musculoskeletal system diseases or bone cancer. About 90% of the population over the age of 40 is susceptible to various types of degenerative diseases, such as arthritis, osteoporosis and bone injuries. Procedures involving the implantation of implants to replace lost, damaged, or deformed tissues under the influence of disease are performed on a mass scale today. This does not mean, however, that the quality of the materials from which individual implants are made has reached perfection. Today, long-term implants (prostheses) and short-term implants (e.g., plates, and bone screws) are used to stabilize broken human bones. The use of short-term orthopedic implants has significantly changed the procedures for treating bone fractures. The materials from which the currently used short-term orthopedic implants are made guaranteed the corrosion resistance of the implant during the period of bone union. After this period, the implant had to be surgically removed. The short-term implants are mainly produced of alloys, which, after fulfilling their functions, must be removed from the human body because they react with tissues and body fluids and thus become toxic to humans [1]. Currently, in the field of orthopedic implantology, resorbable biomaterials are being designed, the degradation products of which can be absorbed by the human body, without adverse health effects. The main barrier to the use of resorbable Mg and Zn alloys is a too high-rate and non-uniform degradation progress. Due to the fact that degradation begins on the surface of the alloy, the most effective method of protection is coating application and obtaining a composite material. In the literature, there are no reports on zinc alloy–polymer composites for medical applications. For the protection of surface zinc alloys, mainly conversion layers were used [2]. In order to observe a corrosion resistance and mechanical strength improvement in zinc alloys, alloy additions were used [3]. Zinc alloys, due to their multi-phase structure, are characterized by a non-uniform degradation process (the degradation process proceeds faster and begins at the interfaces); hence, it is necessary to use a protective coating, e.g., a self-healing casein coating. In this work, the composite material for testing was a zinc alloy ZnMg3.2 wt.% with a self-healing layer composed of casein, NaOH and bone-forming material (nanocrystalline hydroxyapatite). The self-healing phenomenon of the coating is necessary to control the degradation process by gradually filling the coating defects in places where chlorides from human body fluids interfere. In addition, the self-healing capacity of the coating is helpful to compensate for any damage to the coating during the implantation operation. The authors selected the components of the coating bath due to their lack of toxicity to the human body. The main component of the bath is casein, which belongs to a group of milk proteins. In turn, proteins are biopolymers, in which amino acids are monomers [4]. Due to the chemical composition of casein, C (53%), H (7%), O (22%), N (15.65%), S (0.76%), and P (0.85%), it is nontoxic for the human body [5]. The listed elements are also trace elements found in the human body. There are few reports in the literature on the use of casein as a material for coatings for medical implants; Qin et al. [6] confirmed the lack of cytotoxicity of casein and chitosan coatings on a Co-Cr-Mo alloy’s surface. In addition, the authors mentioned probable bioactive properties of this coating, which refers to the increased amount of calcium phosphates on the surface of the samples. Kumar et al. [7] indicated that used casein reduced bone graft porosity and consequently increased the sample’s mechanical properties. The osteogenic material as the second component of the self-healing coating is the carrier of the components necessary for bone reconstruction at the fracture site [8]. The introduction of nanocrystalline hydroxyapatite is intended to accelerate the formation of new bone tissue and ensure osseointegration of the implant with the bone. Nanocrystalline hydroxyapatite (NanoBone^®^) has osteoconductive and osteoinductive properties, which means that it actively stimulates the process of new bone formation and is not just a scaffold [9].

In order to determine the most favorable technological conditions for obtaining self-healing coatings on zinc alloys, the influence of the concentration of casein and NaOH on the progress, quantity and quality of symptoms indicating the occurrence of the self-healing effect of the produced coatings was examined. This work assumes that the self-healing mechanism of the layer will delay the degradation of the substrate by gradually replenishing layer losses in places where chlorides from simulated body fluids interfere, and will also eliminate the effects of possible layer damage during the implantation operation. This study examined the technological conditions and chemical composition of the bath for casein coatings produced on the surfaces of a Zn alloy. Using potentiodynamic tests and microscopic observations, as well as EDS analysis, the impact of the produced layers on the corrosion resistance of the tested alloy was assessed and the probable course of the self-healing phenomenon of the casein-based coating was determined.

## 2. Materials and Methods

The substrate was ZnMg3.2 (wt.%) alloy. The high-purity zinc (99.98%) and magnesium (99.95%) were melted in an induction furnace at 550 °C. For melting and casting, a ceramic crucible (Al_2_O_3_) and protective gas, argon, were used. The master alloy was remelted at approximately 480 °C. The cooling of cast elements took place within the furnace. The sample size is 25 mm in diameter and 5 mm in width. The surface of the samples was sandblasted by Al_2_O_3_ sand of 50 grain size. For EDS analysis, the sample surface was ground on 1200 grid paper and then degreased in acetone. The sample prepared in this way was immersed in the coating bath. The sandblasting step was omitted on purpose not to disturb the EDS chemical composition analysis with the presence of elements from the sandblasted layer. In the aim of preparing self-healing coatings, the substrate samples were immersed in a beaker containing solution with chemical composition and technological conditions given in Table 1.

The solution of casein (produced by Acros Organics, Waltham, MA, USA, pure, https://www.oconchemicals.ie/products/id-199530.html, accessed on 29 November 2023) in distilled water was prepared at a temperature of approx. 40 °C. The sodium hydroxide and nanoHAp were mixed until a homogeneous solution was achieved. The nanocrystalline hydroxyapatite used for research was produced by Nanobone, Saint Cloud, MN, USA (www.nanobone.de). Sodium hydroxide used for the coating bath was produced by Warchem, Warszawa, Poland. In order to determine the most favorable technological conditions to obtained self-healing coatings, the following research plan was devised.

The microstructure of ZnMg3.2 (wt.%) was carried out by light microscopy (Zeiss) and Axiovision Rel. 4.4 software. The structure and effect of the self-healing coating were observed by scanning electron microscopy Supra 35 Carl Zeiss with an EDS analyzer (Jena, Germany). The immersion test to analyze the self-healing effect of coatings was carried out in Ringer’s solution (8.6 g/dm^3^ NaCl, 0.3 g/dm^3^ KCl, 0.48 g/dm^3^ CaCl_2_·6H_2_O) for 1 h at 37 °C. The electrochemical tests were performed by potentiostat Autolab 302 N controlled by NOVA 1.11 software. The potentiodynamic measurements were conducted using a three-electrode cell. AgCl was the reference electrode. Potentiodynamic measurements involved the electrochemical curves determined after 1 h of immersion in Ringer’s solution at 37 °C.

Biological characterization was based on a human osteosarcoma cell line U2OS (cat. No. 92022711; Merck, Rahway, NJ, USA). Cells were grown in Dulbecco’s Modified Eagle Medium/Nutrient Mixture F-12 (DMEM/F12, Merck) supplemented with 10% fetal bovine serum (FBS, Eurx, Gdańsk, Poland) and 1% penicillin/streptomycin (Merck) at 37 °C in constant 80% humidity atmosphere and 5% carbon dioxide concentration (standard conditions for the incubator Heracell™ 150i, Thermo Fisher Scientific, Waltham, MA, USA). The cells were cultivated at a confluence of 60–80%, and then were transferred to the 12-well plates (Sarstedt, Nümbrech, Germany). The UV-sterilized materials, covered by a thin or wide casein-based layer (dropped by automatic pipette in the volume of 500 µL directly on a metal disc) were prepared 24 h earlier, for a good drying process. The material without a casein-based layer was used as a control. The cells were seeded directly on tested materials in the amount of 10 × 10^4^ cells on 1 cm^2^ samples and incubated for 7 days in the completely grown medium. For cell visualization, the fixation procedure with 70% methanol solution (Merck), for 10 min at room temperature, was used. The nucleus of cells was stained using Stain DAPI mounting media (Thermo Fisher Scientific) and visualized by Olympus FluoView FV1000™ confocal microscopy (Olympus LS, Tokyo, Japan).

**Figure 1:**
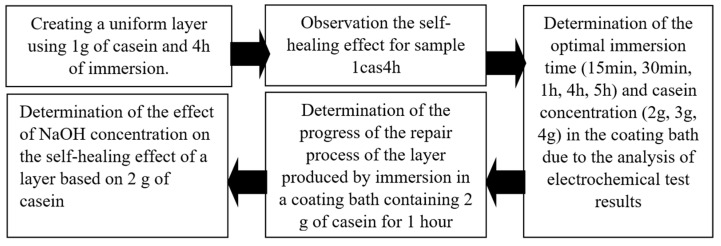
The concept and main goals implemented in this study.

In order to determine the most favorable technological conditions to obtain self-healing coatings, the following research plan was devised (Figure 1).

## 3. Results

**Figure 2:**
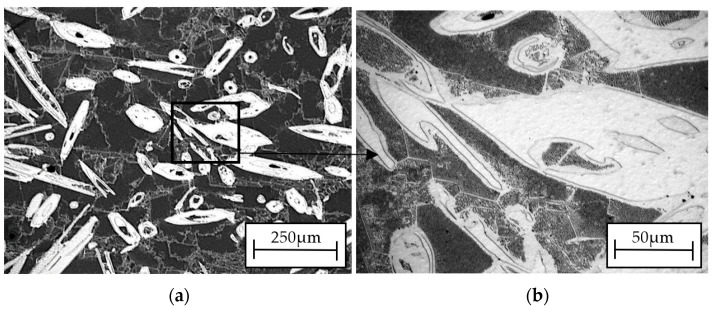
Structure of ZnMg3.2 wt.% alloy at 25× (**a**) and 100× (**b**) magnification.

Analysis of the ZnMg3.2 wt.% alloy (shown in Figure 2) revealed a multi-phase composition. As per the Zn-Mg phase system, this composition encompasses MgZn2 polyhedrons (light) and a Zn + Mg2Zn11 eutectic structure.

**Figure 3:**
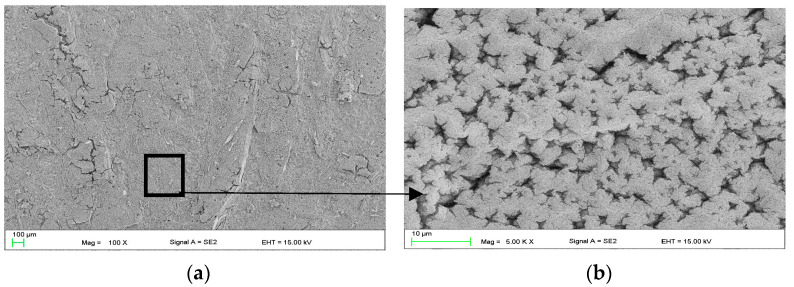
SEM images of casein 1cas4h coating with NanoHAp addition showing surface morphology (**a**) where star-shaped elements may be distinguished at higher magnification (**b**).

As outlined in the established research plan (Figure 1), protective coatings were generated on the ZnMg3.2 alloy surface using the dip-coating method. This process involved immersing the alloy in solutions with varying concentrations of casein and NaOH (Table 1). In the first stage, the coating was evaluated following a 4 h immersion in a dip-coating batch containing 1 g of casein, denoted as 1cas4h (Figure 3). Morphologically, the coating appears quite uniform, showcasing distinct star-shaped elements dispersed across the entire sample surface.

**Figure 4:**
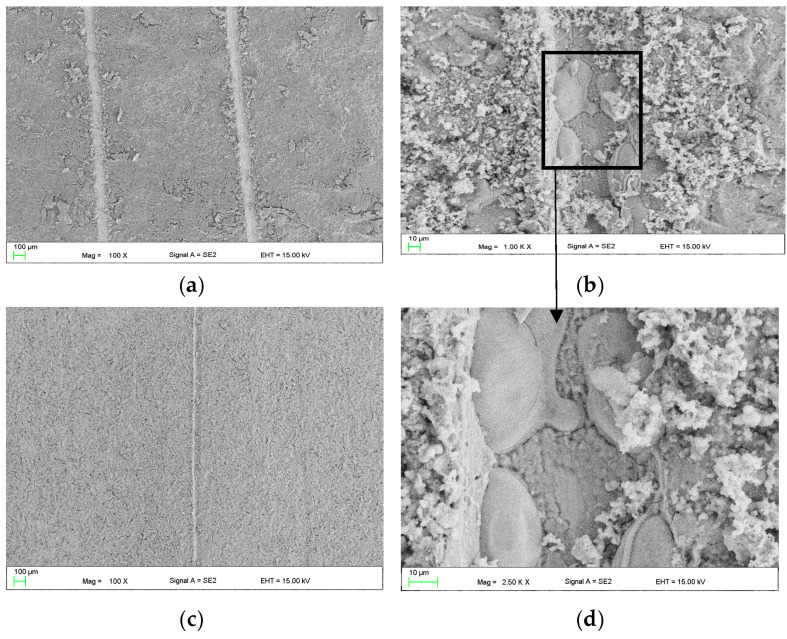
SEM images casein coating with scratch before (**a**) and after (**b**–**d**) immersion in Ringer’s solution at 37 °C of 1cas4h.

Then, the self-healing effect of the coating was assessed. A scratch was made on the 1cas4h sample (Figure 4a). At the edges of the scratch, deformation of the coating and traces of the blade in the middle part of the scratch are visible. A sample with a 1cas4h coating with a scratch was immersed in Ringer’s solution for 1 h, shown in Figure 4b. In order to determine the degree of “repair” of the coating, morphological changes in the area of the intentionally made scratch were examined. SEM observations revealed that the morphology of the scratch surface has changed. The blade traces in the central part of the scratch are invisible. In addition, conglomerates similar to fragments of the shell morphology were observed (Figure 4b). The area with a round-like element is marked in the black frame, suggesting the beginning of the “repair” process of coating.

**Figure 5:**
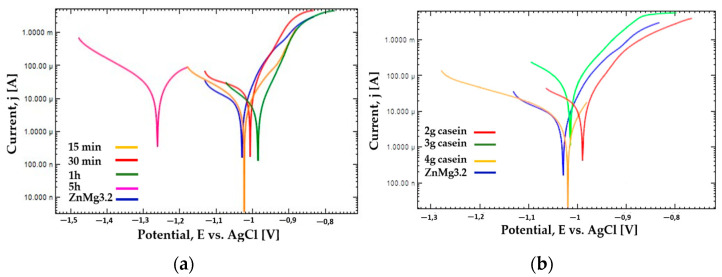
Potentiodynamic curves in Ringer’s Solution at 37 °C for uncoated substrate and samples coated with different immersion times (**a**) and different concentrations of casein (**b**) in the coating bath.

Next, the potentiodynamic corrosion test in Ringer’s solution at 37 °C was performed in order to determine favorable immersion time and casein concentration in the dip-coating baths. Varied concentrations of casein in the coating bath were tested (2 g, 3 g, and 4 g), according to Table 1. The best results were obtained for coatings deposited in bath containing 2 g of casein and 6 g NaOH. For such a concentration of casein (2 g) in the bath, coatings were made for different immersion times in the dip-coating process, i.e., 15 min, 30 min, 1 h and 5 h (samples 2cas15, 2cas30, 2cas1h, 2cas5h). Figure 4 presents the potentiodynamic polarization curves for coatings with 2 g casein addition and varied dip coating immersion time (Figure 5a), and coatings obtained with varied casein addition for the process lasting 1 h (Figure 5b). The potentiodynamic curves of the samples immersed in the coating bath for 15 min, 30 min, and 1 h (Figure 5a) in comparison to the uncoated substrate shift towards positive potentials. Increasing the immersion time to 5 h is unfavorable because the potentiodynamic curve has shifted towards negative potentials compared to the uncoated substrate. Therefore, the optimal immersion time was 1 h of immersion in the coating bath, because the determined potentiodynamic curve shifted most significantly to positive potential values among the tested samples. The potentiodynamic curves of samples submerged in a coating bath containing casein concentrations of 3 g and 4 g (Figure 5b) exhibit a higher potential position to that of the uncoated substrate. Reducing the casein concentration to 2 g is the most favorable because the potentiodynamic curve has shifted toward positive potentials compared to the uncoated substrate.

Table 2 presents E*_corr_* (V), j*_corr_* (µA/cm^2^),R*_p_*(kΩ), and V*_corr_* (mm/year) for the study samples.

The V*_corr_*, j*_corr_* determined from the Tafel analysis is the lowest for samples immersed in a bath with a concentration of 2 g of casein and immersion time in the bath for 1 h. Immersing a Zn alloy sample in a coating bath for 5 h and 30 min is the least advantageous because the values of corrosion current density, polarization resistance and corrosion rate are closest to those values corresponding to the uncoated substrate. The position of the potentiodynamic curves for 5 h and 30 min also indicates low corrosion resistance because they are in a high current range on both the anode and cathode sides compared to the rest of the curves. Similarly, the curves determined for samples immersed for 1 h in a bath with a concentration of 3 g of casein are characterized by the lowest value of polarization resistance and the highest values of corrosion rate and corrosion current density among coated samples, which indicates low corrosion resistance. The position of the potentiodynamic curve determined for the sample coated in a bath with a concentration of 3 g of casein indicates the highest current values on both the anodic and cathodic sides among the tested samples.

**Figure 6:**
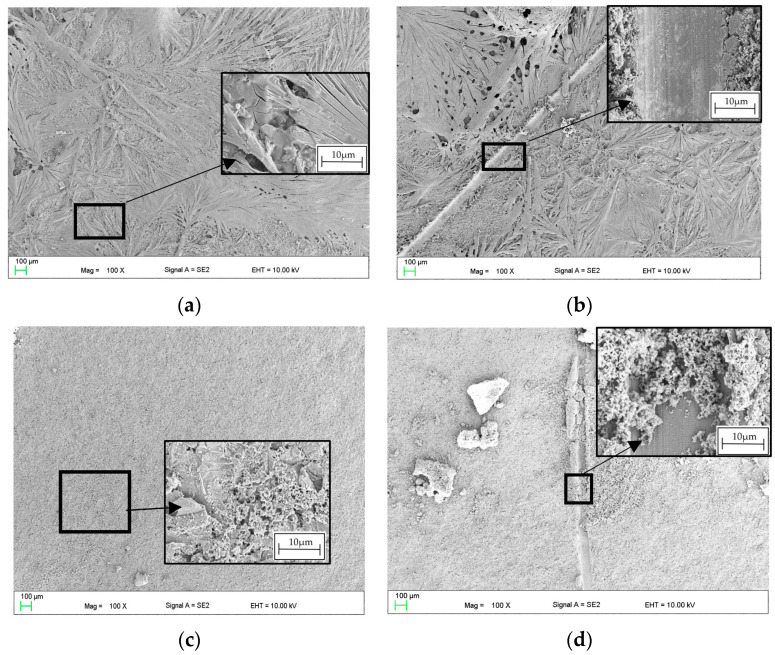
SEM images of casein coating marked as 2cas1h: (**a**,**b**) before and (**c**,**d**) after immersion for 1 h in Ringer’s solution at 37 °C.

Therefore, microscopic examinations were conducted to observe morphological alterations in the region where the intentionally scratched coating made with 2 g of casein addition (2cas1h) was submerged in Ringer’s solution (Figure 6).

**Figure 7:**
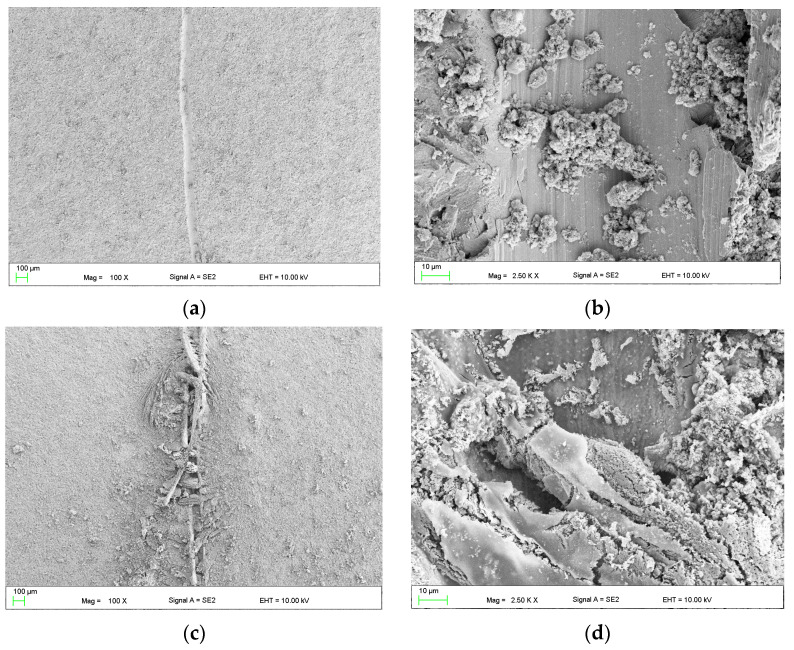
SEM images of casein coating marked as 2naoh2cas (**a**,**b**) and 4naoh2cas (**c**,**d**) after 1 h of immersion in Ringer’s solution at 37 °C.

The coating’s morphology is defined by crystal-like branches that create a cohesive layer on the surface of the substrate (Figure 6a). The scratch evident in Figure 6b displays blade traces in the center and coating deformation at the edges. After an hour-long immersion in Ringer’s solution at 37 °C, the casein layer in Figure 6c exhibits enhanced surface development. Notably, within the scratch area in Figure 6d, there is an almost “repaired” appearance, featuring elements resembling the structure of the coating found outside the scratched region. To assess the impact of NaOH concentration on the self-healing capabilities of the 2 g casein-based layer, coating baths were formulated with 2 g NaOH (2naoh2cas) and 4 g NaOH (4noah2cas). Subsequently, samples were immersed in these baths for 1 h. Following drying, the coated samples underwent a 1 h immersion in Ringer’s solution at 37 °C. The observed outcomes regarding the self-healing effect of the layer after this hour-long immersion in Ringer’s solution are presented in Figure 7. Lowering the concentration to 2 g of NaOH (Figure 7a,b) in the coating bath does not produce the self-healing effect. No notable indications suggestive of the layer self-repairing were observed. Conversely, a layer formed in a bath with 4 g of NaOH exhibits significantly improved self-healing effects. Distinctive overhangs and even “ribs” connecting both edges of the crack (Figure 7c,d) are visible, likely resulting from material sourced from the coating and subsequent reaction with the solution.

**Figure 8:**
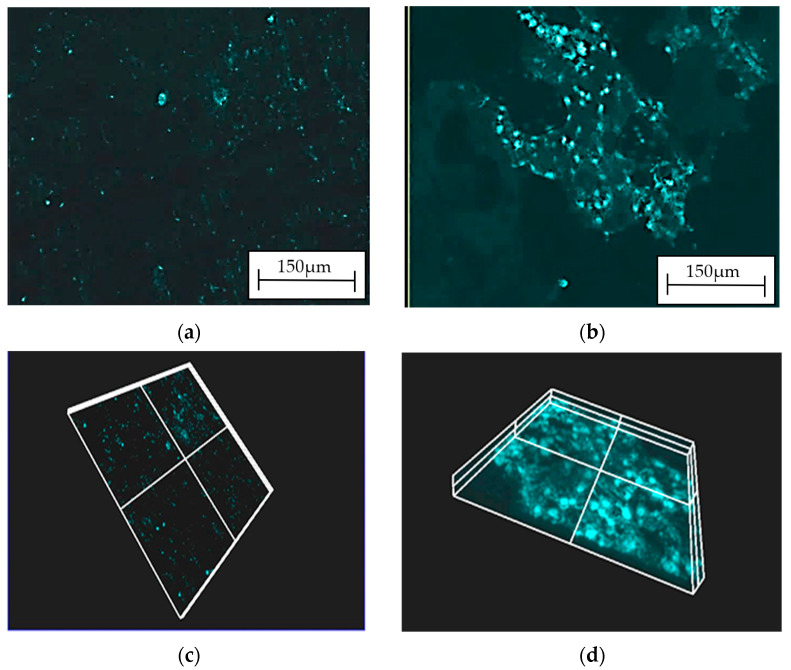
Confocal images of U2OS cells with DAPI-stained nucleus after 7 days of incubation on casein-based layer (2cas1h) on tested materials: ZnMg3.2 alloy (**a**); casein coating (2cas1h) (**b**). Additional 3D visualization from Immaris software (Bitplane Scientific Software; version 10.0.0; Olympus, Zurich, Switzerland) for ZnMg3.2 alloy (**c**), casein coating (2cas1h) (**d**).

The biocompatibility of the produced casein-based coatings was evaluated through a biocompatibility assay conducted with osteosarcoma cells (Figure 8).

Optimal cellular adhesion and proliferation for U2OS osteosarcoma cells were observed after 7 days of incubation on a casein coating (2cas1h) (Figure 8b,d). This signifies that the alloy surface, when covered by the casein layer, significantly enhances cell adhesion. In contrast, uncoated ZnMg3.2 alloy exhibits notably poor cell survival (Figure 8a,c). Cells struggled to proliferate on this substrate due to its surface, which does not support optimal adhesion for this specific cell line (U2OS being an adherent cell type).

**Figure 9:**
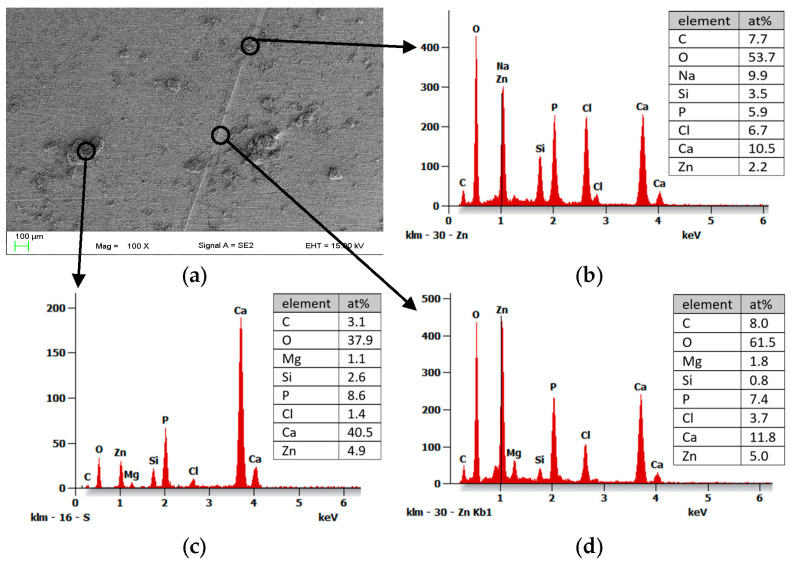
Results of EDS chemical composition tests of a sample 2cas1h without sandblasting after an hour of immersion in Ringer’s Solution at 37 °C. The analyzed surface area (**a**) and chemical composition of specific microregions (**b**–**d**).

In order (Figure 9) to examine the qualitative chemical composition of the elements found in the scratch area after an hour of immersion in Ringer’s solution, tests were carried out using an EDS analyzer on a sample with a casein coating, but without a sandblasted substrate.

In the location of the scratch, along with visible agglomerates on the edges and in the middle (Figure 9b), P, and Ca, which contains hydroxyapatite in its composition, were detected. In turn, the presence of chlorine most likely comes from the Ringer solution. Silicon is rather an impurity from the dip-coating process or constituent elements. The result of EDS analysis also indicated the presence of a large amount of zinc (Zn), which comes from the substrate and to a large extent may also come from zinc oxide, which is a transformation product of Zn(OH)_2_ produced in chloride environments [10]. The clusters visible in the coating are probably coagulated nanoHAp powder, judging by the high peak from Ca (Figure 9c). The EDS result shown in Figure 9d, in turn, is from the crack area in which there are no such significant coagulations and agglomerates. The presence of analogous elements, as those observed in the EDS data from Figure 9b, was identified. With the exception of one sample, the presence of magnesium (Mg) was detected. Additionally, the zinc (Zn) peak appeared notably higher. This may indicate an uncovered substrate, uncoated or partially coated. Another potential explanation for the presence of magnesium (Mg) in the EDS results could be the existence of a eutectic or another structural phase within this specific area of the substrate.

**Figure 10:**
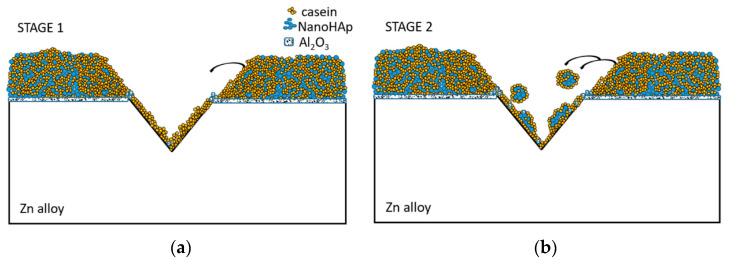
The proposal of stages of the casein-nanoHAp self-healing effect in the first stage (**a**) and second stage (**b**).

Based on the findings of this study, it can be concluded that there is a self-healing effect observed in the casein-based layers. The mechanism of this phenomenon is presented graphically in Figure 10. This study’s results reveal a two-stage self-healing mechanism observed in the casein-based layers investigated. The first stage likely involves the hydrolysis of casein, resulting in its infiltration into the scratch. This is observable as round elements within the scratched area and overhangs along the scratch’s edges. Stage 2 involves the migration of nanoHAp particles, bound within the casein, toward the scratched area. This process leads to the formation of “ribs” that connect the edges of the scratch. Both stages probably occur simultaneously, only to a different extent, in different scratch areas. It seems that nanoHAp particles play two roles: as a component of the self-healing layer and as a reservoir of casein as a healing agent. On the other hand, casein also has two roles: as a component of the healing film and as a courier of nanoHAp molecules as healing ingredients. Therefore, these materials play complementary roles, making the nanoHAp-casein coating self-healing.

**Figure 11:**
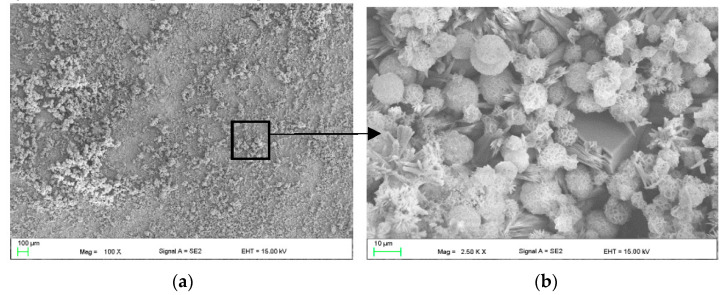
SEM images of casein 2cas1h sample after one week immersion in Ringer’s solution at 37 °C showing surface covered with corrosion products (**a**), where spherical and irregular forms compose a very tight layer of corrosion products (**b**).

The sample with the 2cas1h coating was immersed for a week in Ringer’s Solution at a temperature of 37 °C (Figure 11). Both spherical and irregular elements form a very tight layer of corrosion products. (Figure 11b).

## 4. Discussion

In this work, self-healing layers were produced on the ZnMg3.2 wt.% alloy. This alloy was chosen due to its potential use as a short-term implant material. In the literature on the subject, it is given as an alloy that meets the criterion of strength properties capable of carrying loads during operation as an orthopedic implant [11]. According to the literature, its tensile strength is approximately 291 MPa [12], which is a value similar to the Magnezix alloy, already used as an implant material [13]. Structure observations (Figure 2) of the substrate alloy for self-healing layers confirms its multiphase structure, which could result in significant damage to a zinc alloy implant shortly after implantation. A similar structure was reported in [10] where MgZn2 polyhedrons and Zn + Mg2Zn11 eutectic were present. The appearance of the above structure was treated as a good basis for protecting the surface against heterogeneous degradation in simulated body solutions. The self-healing coatings produced in this work actually change the course of the corrosion of zinc alloys. The casein coating not only prevents the heterogeneous progression of corrosion of zinc alloys, but also eliminates micro-damages that are inevitable during implantation surgery. This is a very important aspect that is often overlooked in studies of resorbable alloys coated with coatings that do not have a self-healing effect. Moreover, introduction of the interlayer as a sandblasted surface unifies the surface and slightly increases its roughness, improving the adhesion of the self-healing layer based on casein and nano HAp. Self-healing coatings as a corrosion barrier with a function of bioactive support of osseointegration of the implant with bone as a solution for inert biomaterials, such as stainless-steel orthopedic implants, were studied by Katta et al. [14]. The authors showed that a self-healing Ce and Nb oxide coating on 316 SS stainless steel does not exhibit cytotoxicity. However, the self-healing coating on Zn alloys acts as a potential biomaterial for resorbable short-term implants is a new topic in materials science. In the literature, there are several reports on self-healing coatings for medical applications, but only on Mg-based industrial alloys [15,16]. In this work, the self-healing effect of dip-coated ZnMg3.2 alloys was studied, where dip coating bath was composed of a varied content of casein (1 to 4 g), NaOH (2 to 6 g) and 0.1 g of nanoHA in distilled water and the duration of the dip-coating process. The coating performance was evaluated in potentiodynamic corrosion tests. Such a method was already used by Zeng et al. [17] to explain self-healing mechanisms of molybdate-based coating on AZ31 alloy. The successive stages of self-healing were observed as a stepwise change in current value on the anodic side. In addition, the corrosion potential of the self-healing layer increased to positive values compared to that of the uncoated sample [17]. In this work, no abrupt changes in the current value occurred on the anode curves. However, this study revealed notable variations in corrosion potential, indicating the impact of specific factors of the dip-coating method like immersion time (Figure 5a) and casein concentration (Figure 5b) on the corrosion resistance of coated samples. The sample coated with 2 g of casein for 1 h displayed the potentiodynamic curve closest to the positive potentials. Samples coated for 15 and 30 min also moved towards positive potentials. However, the potentiodynamic curves of these samples were similar to the uncoated sample’s corrosion potentials. This indicated that the casein layers were thin and degraded after an hour, causing the potential to correspond more to the substrate than the casein coating. Therefore, the extent of layer repair depends on technological factors such as the concentration of casein and the immersion time of substrate in the coating bath. Scanning microscopy proved to be a valuable method for evaluating the self-healing effect of coatings and assessing the progress of repair [18]. In the present study, depending on the immersion time applied, the repair mechanism occurred in a different range. Based on microscopic examinations, the most advantageous condition was 1 h of immersion in the coating solution with 2 g of casein in the coating bath, as it resulted in the largest range of coating repair. Comparing the morphology of the layer immersed for 4 h (Figure 4) with that immersed for only 1 h (Figure 6), significant differences in structure were observed, likely influencing the degree of repair coating. It was noticed that although the layers obtained with 1 g of casein and 4 h of immersion time (Figure 4) exhibited a uniform surface structure, they contained round gaps that allowed chlorides from the Ringer solution to penetrate easily. The coating in Figure 6, due to its more compact structure, does not have visible defects through which chlorides could penetrate. When comparing the surface of the samples after removal from the coating baths after 1 h and 4 h, the differences were more visible. The sample that stayed in the coating bath for 4 h looked slightly etched. This is the reason that created defects in the final coating structure shown in Figure 3. However, during immersion in the bath, an effect of etching the surface of the sample was observed. Therefore, more of the alloy surface was exposed (etched) than covered with casein. Therefore, it was decided to increase the casein concentration in the bath to 2 g and check whether such a long immersion time would actually result in uneven coating coverage and, consequently, lower corrosion resistance. The results of the potentiodynamic tests confirmed this thesis. In Figure 5b, the immersion time of 5 h shifted the potential of the tested samples to the greatest extent towards lower potentials and, consequently, lower corrosion resistance. It was found that this was related to the observed etching of the alloy surface during immersion in the coating bath. On the basis of the results of this study, it can be concluded that there is a self-healing effect in the casein-based layers. The probable repair mechanisms of the layers obtained are similar for both samples. Mechanisms of casein degradation have not yet been investigated, but it is worth emphasizing that casein is a group of phosphoproteins that have the ability to bind water molecules. Therefore, in aqueous solutions, phosphoproteins swell and then dissolve to form colloidal particles. The products of these reactions were probably visible in SEM images on Figure 4b, Figure 6d and Figure 7c,d. However, the extent and progress of “repairing” the layer depends on the production technology, and, in particular, on the immersion time and concentration of NaOH in the coating bath. The sample, which was immersed in the coating bath for 4 h after the Ringer solution immersion test, showed morphological changes that indicated a gradual accumulation from clusters of products of the reaction of the coating with the Ringer solution. The element marked in the frame in Figure 3b resembles the pouring of liquid, perhaps casein, into the middle part of the scratch. However, in the area of the crack on the sample immersed for 1 h in the coating bath, morphological changes are visible, indicating more overgrowth in the products of the reaction of the layer with Ringer’s solution at both the edges and the middle part of the crack. The result of this process is the disappearance of a deliberately made scratch. In turn, Figure 7c,d show almost complete regeneration of the scratch. There are visible overhangs on the edges of the scratch and “ribs” connecting both edges of the scratch. Reducing the concentration of NaOH in the coating bath to 4 g made it possible to obtain the most visible effects in the form of “overgrowing” of the scratch with the coating material and what was the result of the reaction of the coating with Ringer’s solution. Taking into account that the concentration of each reagent used to prepare the coating bath is important, it was found that for the NaOH concentration used in the case of samples with 3 g and 4 g of casein, it was ineffective. The authors indicate that the NaOH concentration is crucial for the self-healing effect. Figure 7 shows the results of observing the self-healing effect for a coating produced in a bath consisting of 2 g of casein, 2 g of NaOH and 4 g of NaOH. Reducing the concentration to 2 g of NaOH (Figure 7a,b) in the coating bath does not result in a self-healing effect. No significant symptoms indicating self-healing of the layer were observed. On the other hand, a layer formed in a bath containing 4 g of NaOH results in much better self-healing effects than for 6 g of NaOH. Therefore, it can be said that, due to the fastest achievement of self-healing of the layer, the concentration of 2 g of casein and 4 g of NaOH in the coating bath is optimal. The casein-based layers on metal alloy improved the cells’ adhesion and proliferation rate, tested in experimental model osteosarcoma U2OS cells, during 7 days of live biocompatibility assay. Confocal images showed a cover-wide dimension dependence—the cells grew up in the casein-based layer(2cas1h). As a concluding remark, the thin casein-based layer displays a promising future to be an organic component for metal implant covers and for cell culturing in vitro. The enhanced biocompatibility stems from the presence of organic components on the metal surface, promoting favorable conditions for cell survival and proliferation.

Taking into account the physicochemical properties of casein such as the binding of ions and small molecules, highly emulsifying and self-organizing properties along with the ability to gel and bind water [19], it is assumed that the casein layer is directly related to the layer visible in SEM images on Figure 11 or even products from its degradation are related to ions and small particles from Ringer’s solution and Zn alloy ions. Taking into account also the biocompatibility tests of the 2cas1h sample, it is assumed that the course of degradation and what will be formed on the surface of the tested coated alloy will be strongly related to the bioactivity of casein, which is widely described in the literature [6,7,20,21]. Scientists often indicate casein as a drug carrier due to its biodegradability and bioactive properties [21]. The authors indicate in the results that the tested casein–chitosan coatings do not show cytotoxicity [6]. However, they indicate the bioactive properties of the tested coatings, consisting in the increased amount of calcium phosphates on the surface of the samples. In turn, Kumar et al. in their research [7] used casein as a material for bone-tissue-engineering purposes (material for bone grafts). The use of casein reduced the porosity and thus increased the mechanical properties of the bone graft. In ref. [21], casein was also used as a component of nanogel for the treatment of skin cancer. The sample with a casein coating (2cas1h) improves the biocompatibility of the zinc alloy, including better cell response. Casein, as a natural food product, is a GRAS protein (generally recognized as safe), and is biocompatible and biodegradable. Casein has low water solubility, but the addition of NaOH in the coating bath causes it to hydrate and acquire hydrophilic properties, which has a beneficial effect on its biocompatibility. Generally speaking, proteins are good raw material because they have the advantages of absorption and low toxicity of the final degradation products [22]. Taking into account the physicochemical properties of casein such as binding of ions and small molecules, there is a possibility that casein decomposition products combine with Zn and Mg ions, which also have a positive effect on cell viability.

## 5. Conclusions

The material developed, comprising a polymer self-healing layer based on casein and nanoHAp combined with a metallic Zn alloy, demonstrates potential as a barrier against uneven or extensive degradation in metal biomaterials. This study verifies that the formulated coating baths yield materials capable of generating self-healing coatings suitable for medical applications. Notably, the components used in these coating baths, such as casein, nanoHAp, or NaOH, lack highly toxic ingredients, making them well-suited for implantology applications.

The work identified optimal concentrations of 2 g casein and 4 g NaOH, along with an immersion time of 1 h for potential implant materials in the coating bath. These parameters were determined to enhance corrosion resistance and achieve an effective degree of “repair” for deliberately induced layer damage.

Taking into account the application perspective, understanding the examined repair mechanisms and their degree is crucial, considering the potential use of this material as a biomaterial for resorbable orthopedic implants. The environment that implants encounter—body fluids and human tissues—is highly demanding. Compensating for microdamage, even before implantation, through the self-healing effect of the layer becomes crucial. Developing a robust coating with self-healing properties against potential damage serves as an active protective measure in this proposed solution.

## Figures and Tables

**Table 1:** Process conditions of dip batch coating and their designations.

Coating Bath Chemical Composition				Immersion Time	Designation of Sample
Casein (g)	NaOH (g)	nanoHA	Rest		
1 g	6 g	0.1 g	distilled water	4 h	1cas4h
2 g				15 min	2cas15
				30 min	2cas30
				1 h	2cas1h
				5 h	2cas5h
3 g				1 h	3cas1h
4 g				1 h	4cas1h
2 g	2 g			1 h	2naoh2cas
2 g	4 g			1 h	4naoh2cas

**Table 2:** Results of Tafel’s analysis for studied samples.

**Sample (2 g Casein in Coating Bath)**	**E*_corr_* (V)**	**j*_corr_* (µA/cm^2^)**	**R*_p_* (kΩ)**	**V*_corr_* (mm/year)**
15 min	−1.02	9	2.5	0.22
30 min	−1.01	18	0.8	0.43
1 h	−0.98	6	2.5	0.15
5 h	−1.26	32	1.08	0.75
ZnMg3.2	−1.03	40	2.2	0.93
**Sample (1 h of Immersion in Coating Bath)**	**E*_corr_* (V)**	**j*_corr_* (µA/cm^2^)**	**R*_p_* (kΩ)**	**V*_corr_*(mm/year)**
2 g casein	−0.98	6	2.5	0.15
3 g casein	−1.01	62	0.3	1.46
4 g casein	−1.02	8	3.2	0.20
ZnMg3.2	−1.03	40	2.2	0.93

## Data Availability

Data are contained within the article.
